# Systematic Evaluation of the Nutritional Quality, Elemental Safety, and Preventive Effects of Perilla Seed Oil on Hyperlipidemia and Gut Microbiota Dysbiosis in High-Fat Diet-Fed Rats

**DOI:** 10.3390/nu18132149

**Published:** 2026-07-02

**Authors:** Jianfeng Chang, Peng Hu, Peiyi Zhang, Xue Yang, Peiyan Ai, Junjie Wei, Leyuan Li, Lianzhen Li

**Affiliations:** College of Agronomy, Henan Agricultural University, Zhengzhou 450046, China; changjianfeng@henau.edu.cn (J.C.); h931702818@163.com (P.H.);

**Keywords:** perilla seed oil, nutritional assessment, acute toxicity, high-fat diet, gut microbiota

## Abstract

**Background:** Perilla seed oil (PSO) is a high-nutritional-value oil and widely used in functional foods, and derives from the mature seeds of *Perilla frutescens*. This study aimed to systematically evaluate the nutritional characteristics and safety of PSO, as well as to investigate its lipid-modulating effects and the underlying changes in gut microbiota in hyperlipidemic conditions. **Methods:** The nutritional characteristics of PSO (prepared via seed cleaning, cold-pressing, filtration, and solvent extraction) were evaluated by comparing it with 15 representative vegetable oils, focusing on fatty acid composition, total phenolic and flavonoid contents, metal elements, and physicochemical indices. The safety of PSO was assessed through acute oral toxicity testing in Kunming mice (doses: 2.5, 5, 10 g/kg) with general observations, histopathological examination, and serum biochemical analysis. Additionally, a high-fat diet (HFD)-induced hyperlipidemic Sprague-Dawley (SD) rat model was established to explore PSO’s lipid-modulating effects and its regulatory role in gut microbiota, using serum biochemical detection, liver pathology examination, 16S rRNA gene sequencing, and short-chain fatty acid (SCFA) analysis. **Results:** PSO possessed the highest α-linolenic acid (ALA) content among the tested oils, along with a favorable unsaturated fatty acid ratio. Notably, PSO was rich in zinc and free of toxic elements. In HFD-fed rats, 10 g/kg PSO significantly reduced serum total cholesterol (TC) and low-density lipoprotein cholesterol (LDL-C) levels, increased high-density lipoprotein cholesterol (HDL-C), and alleviated hepatic damage. Moreover, PSO modulated gut microbiota by enriching probiotic populations and elevating intestinal production of short-chain fatty acids (SCFAs), particularly propionate and butyrate. **Conclusions:** PSO is a nutritionally rich and safe edible oil with notable lipid-modulating properties, highlighting its potential as a dietary intervention for preventing lipid metabolism disorders.

## 1. Introduction

The global demand for vegetable oils is very high. Among the various oils, palm oil, soybean oil, and rapeseed oil dominate the world market due to their high yields, substantial oil production rates, and strong stability [1]. In addition, medicinal and edible homologous oils have gradually attracted attention and are being used as health-care products because of their rich beneficial chemical compositions. Thus, the production, import, and export of these oils have also shown a steady growth trend.

PSO constitutes approximately 40% to 50% of the perilla seed content, which is derived from the annual medicinal and edible homologous herb of *Perilla frutescens* [2]. PSO had a global market size of USD 874.9 million in 2021, with projections of expanding steadily; this also reflects the promising market prospects for it. The modern nutritional perspective redefined PSO as a high-value functional lipid in the 1970s, driven by the discovery of over 55% α-linolenic acid (ALA, C18:3 *n*-3) content [2]. As a core member of the omega-3 polyunsaturated fatty acid (PUFA) family, ALA is an essential dietary nutrient that exerts cardiovascular protection through multi-target mechanisms [3,4], including lipid metabolism regulation [5], anti-inflammatory activity [6], and vascular endothelial function improvement [7].

Dietary intervention has been shown to be an effective strategy for regulating lipid metabolism. Recent studies showed that PSO is rich in various effective ingredients, including fatty acids [8,9], flavonoids, phenolic acids, ferulic acid and vanillic acid, which act synergistically to enhance its nutritional value and health-promoting properties [10,11]. These ingredients exhibit a myriad of benefits, notably enhancing intestinal health and helping to manage metabolic diseases. Despite the preliminary evidence supporting the fact that incorporating PSO into a diet can effectively increase the abundance of beneficial bacteria in the gut microbiota [12], the relationships with the gut microbiota and lipid metabolism remain poorly characterized. Moreover, most studies focus on extraction optimization [2,13,14] or component analysis [15,16], lacking comprehensive nutritional and safety comparisons with widely used vegetable oils.

To address these gaps, this study systematically evaluated the nutritional advantages and safety profile of PSO through multidimensional analyses of fatty acid profiles, bioactive constituents, trace metal composition, and physicochemical parameters. Furthermore, we employed an acute oral toxicity assay and a HFD-induced rat model to elucidate the effect of PSO on lipid metabolism modulation and gut microbiota regulation. These findings aimed to provide scientific evidence for PSO’s application as safe and functional edible oil.

## 2. Materials and Methods

### 2.1. Oil Samples and Materials

PSO was prepared following the previously described method [17], which involved seed cleaning, cold-pressing, filtration to remove impurities, and extraction purification. The detailed procedure was as follows: perilla seeds were mechanically pressed at a temperature below 45 °C, and the pressed liquid was collected and filtered. An equal volume of 10% saline solution at 60 °C was added to the filtrate, followed by thorough stirring and static extraction for 12 h. Finally, PSO was obtained by separation. The following edible oils were obtained from their respective manufacturers: palm oil (PO; Henan Fanglin Food Technology Co., Ltd., Zhengzhou, Henan, China), sesame oil (SO; Laiyang Luhua High-end Edible Oil Co., Ltd., Laiyang, Shandong, China), walnut oil (WO; Shaanxi Guanzhong Oil Workshop Oil Co., Ltd., Xi’an, Shaanxi, China), avocado oil (AO; Liaoning Shengmai Industrial Co., Ltd., Dalian, Liaoning, China), flaxseed oil (FO; Jiangsu Jinzhou Grain and Oil Food Co., Ltd., Nanjing, Jiangsu, China), tea seed oil (TSO; Hunan Xinjinhao Tea Oil Co., Ltd., Changsha, Hunan, China), grape seed oil (GSO; Shanxi Xinjinshang Biotechnology Co., Ltd., Taiyuan, Shanxi, China), sunflower oil (SunO; Anhui COFCO Oil Co., Ltd., Hefei, Anhui, China), olive oil (OO; Shanghai Chenjia Food Technology Co., Ltd., Shanghai, China), soybean oil (SBO; Yihai Kerry Grain and Oil Industry Co., Ltd., Shanghai, China), corn oil (CO; Yihai Kerry Grain and Oil Industry Co., Ltd., Shanghai, China), blended oil (BO; Yihai Kerry Food Marketing Co., Ltd., Shanghai, China), rice bran oil (RBO; Shanghai Kerry Food Industry Co., Ltd., Shanghai, China), peanut oil (PeaO; Shandong Luhua Group Co., Ltd., Laiyang, Shandong, China), and rapeseed oil (RO; Yihai Kerry Grain and Oil Industry Co., Ltd., Shanghai, China).

### 2.2. Animal Experiment Materials

A total of 40 Kunming mice (20 females and 20 males, weighing 20–22 g, five weeks old) and 50 male Sprague-Dawley (SD) rats (weighing 200–220 g, six weeks old) were purchased from Liaoning Changsheng Biotechnology Co., Ltd. (Benxi, Liaoning, China) (Animal Production License No. SCXK (Liao) 2025-0002). All animals were housed in the specific pathogen-free (SPF) facility of the college and handled in compliance with the Care and Use of Laboratory Animals guidelines, with approval from the Henan Agricultural University Animal Ethics Committee (Approval No. HNND2025110601). For the animal experiments, the basal feed was purchased from Beijing Keao Xieli Feed Co., Ltd. (Beijing, China), containing corn, soybean meal, fish meal, wheat flour, yeast powder, vegetable oil, and salt, as well as a variety of vitamins and mineral elements. The HFD was formulated to contain 63.6% basal feed, 20% sucrose, 15% lard, 1.2% cholesterol, and 0.2% sodium cholate (by weight), in total containing 14.67% protein, 23.74% fat, 61.39% carbohydrates and 0.2% sodium cholate.

### 2.3. Fatty Acid Composition Analysis

Following the established sample preparation method [17], all vegetable oils were processed to convert the fatty acids into their corresponding methyl esters. The quantitative analysis of fatty acid methyl esters was performed using a Shimadzu GC 2010 PLUS gas chromatograph system (Shimadzu Corporation, Kyoto, Japan) equipped with a flame ionization detector and SH-Rtx-Wax column (30 m × 0.25 mm × 0.25 μm, Shimadzu Corporation, Kyoto, Japan). The injector and flame ionization detector temperatures were both set at 250 °C. The column temperature was programmed as follows: initial hold at 50 °C for 1 min, then increased to 200 °C at a rate of 25 °C/min, further raised to 230 °C at 3 °C/min and held for 20 min. For analysis, individual fatty acids were categorized into unsaturated fatty acids (UFAs) and saturated fatty acids (SFAs). Among UFAs were palmitoleic acid (C16:1), oleic acid (C18:1), linoleic acid (C18:2), α-linolenic acid (C18:3), and eicosenoic acid (C20:1). SFAs contained lauric acid (C12:0), myristic acid (C14:0), palmitic acid (C16:0), stearic acid (C18:0) and arachidic acid (C20:0). The contents of these fatty acids in the various vegetable oils were quantified using the external standard method.

### 2.4. The Determination of Total Phenolic and Total Flavonoid Content

The total phenolic content in vegetable oils was determined using the Folin–Ciocalteu colorimetric method. Briefly, 1.0 g of oil sample was extracted with 80% (*v*/*v*) methanol. The extract was then reacted sequentially with Folin–Ciocalteu reagent and 7.5% (*w*/*v*) sodium carbonate solution. After incubation under appropriate conditions, the absorbance of the resulting mixture was measured at 765 nm using a UV-1900 UV-Vis spectrophotometer (Shanghai Jingqi Instrument Co., Ltd., Shanghai, China).. A calibration curve was constructed using gallic acid as the standard (concentration range: 0–60 µg/mL). Results were expressed as micrograms of gallic acid equivalents (GAE) per gram of oil. Total flavonoid content was assessed by the aluminum nitrate colorimetric method. A 0.5 g aliquot of oil sample was mixed with 10 mL of 70% (*v*/*v*) ethanol and subjected to ultrasonic-assisted extraction for 30 min. The supernatant was collected and mixed successively with 5% (*w*/*v*) sodium nitrite, 10% (*w*/*v*) aluminum nitrate, and 4% (*w*/*v*) sodium hydroxide solutions. The absorbance of the final reaction mixture was recorded at 510 nm. A standard curve was prepared using rutin (concentration range: 0–100 µg/mL), and the total flavonoid content was expressed as micrograms of rutin equivalents per gram of oil.

### 2.5. Metal Element Composition Analysis

Na, Mg, K, Ca, Mn, Fe, Ni, Cu, Zn, As, Cd, and Pb contents of each oil sample were determined using inductively coupled plasma optical emission spectrometry (ICP-OES) after microwave digestion. Briefly, 1.0 g of oil sample and 3 mL of 75% HNO_3_, prepared by diluting concentrated 70% HNO_3_, were added into microwave digestion tubes and placed into an EXPEC 790S super microwave digestion workstation (EXPEC Technology, Hangzhou, China). The samples were digested at 240 °C under a pressure of 4 MPa for 20 min and then cooled to room temperature. After filtration through a 0.22 µm membrane, metal ion analysis was performed using an EXPEC 6100 ICP-OES (EXPEC Technology, Hangzhou, China), following the previous methods [18]. Instrument operating parameters: RF power was 1450 W, cooling gas was 13 L/min, nebulizer gas was 0.55 L/min, auxiliary gas was 0.8 L/min, analytical wavelength was 267.72 nm, integration time was 15 s, the nebulization chamber temperature was 6 °C, and the peristaltic pump speed was 20 r/min.

### 2.6. Determination of Acid Value, Iodine Value, Peroxide Value, and Saponification Value

The acid value (AV) was calculated using the method of GB 5009.229-2025 [19], and the iodine value (IV) was determined by the protocol based on GB/T 5532-2022 [20]. The saponification value (SV) was determined by GB/T 5534-2024 [21]. The peroxide value (PV) of oleogel was determined using the methods outlined in the Official methods GB 5009.227-2023 [22].

### 2.7. Acute Oral Toxicity Testing of PSO

#### 2.7.1. Animal Grouping and Administration

Forty mice (20 males and 20 females) were randomly assigned into four groups (*n* = 10): a control group and three treatment groups receiving PSO at doses of 2.5, 5, or 10 g/kg. Males and females were housed separately. All animals were fasted for 12 h prior to dosing with free access to water. After a single oral gavage dose, the mice were closely monitored for the first 6 h after administration and subsequently observed once or twice daily for 7 consecutive days. All surviving animals were fasted overnight (for about 12 h) on the last day of the observation period before the collection of blood samples. The liver, spleen, and kidney tissues were harvested for subsequent analyses.

#### 2.7.2. General Observations

Throughout the 7-day period, general health parameters, including food intake, physical appearance, behavior, secretions, excretions, signs of toxicity, and mortality, were systematically recorded. Any animal found dead or moribund was immediately necropsied, and gross pathological changes in major organs, including gastric tissue, intestines, liver, and kidneys, were documented.

#### 2.7.3. Histopathological Examination

Liver and kidney tissues were collected, fixed in 4% paraformaldehyde, dehydrated, embedded in paraffin, and sectioned at 4 µm thickness. The tissue sections were stained with hematoxylin and eosin (H&E) and the histopathological changes were observed under an optical microscope at 200× magnification.

#### 2.7.4. Serum Biochemical Analysis

Serum samples were collected after centrifugation at 3000 rpm for 15 min, and the supernatant was carefully aspirated and stored at −80 °C. The serum levels of alanine transaminase (ALT), aspartate transaminase (AST), blood urea nitrogen (BUN) and creatinine (Cr) were determined using corresponding kits purchased from Nanjing Jiancheng Bioengineering Institute (Nanjing, Jiangsu, China), in accordance with the manufacturer’s instructions. The batch numbers of each kit were as follows: ALT (C009-2-1), AST (C010-2-1), BUN (C013-2-1), and Cr (C011-2-1).

### 2.8. In Vivo Pharmacodynamic Experiments of PSO

#### 2.8.1. Hyperlipidemia Modeling Establishment

Following a one-week acclimatization period, fifty rats were randomly assigned to five groups: control group, model group, PSO-L group, PSO-M group, and PSO-H group (*n* = 10 per group). Subsequently, the normal control group was given a standard diet, while the other groups were given a HFD to construct hyperlipidemia rat models. The control and model groups received daily intragastric administration of distilled water, while the PSO-L, PSO-M, and PSO-H groups were given different doses of 5, 10, and 15 g/kg/d PSO according to the preliminary experiment in rats, and both HFD-induced modeling and treatment groups were administered concurrently with gavage twice a day for 8 weeks. All animals survived during the modeling and dosing period. After 8 weeks of taking PSO, the animals were fasted overnight for about 12 h on the last day of the experimental model, and induced with 1% pentobarbital sodium for anesthesia. The ileal contents were aseptically collected, transferred into 5 mL microcentrifuge tubes, immediately snap-frozen in liquid nitrogen, and stored at −80 °C until further analysis. Liver tissues were dissected and fixed in 4% paraformaldehyde for 48 h for H&E analysis.

#### 2.8.2. Physicochemical Index Analysis

The cholesterol (TC) and triglyceride (TG) levels were determined using an automatic biochemical analyzer (Indiko, Thermo Fisher Scientific, Waltham, MA, USA) according to the manufacturer’s instructions.

#### 2.8.3. H&E and Oil Red O Staining Analysis

The H&E staining analysis of the liver was conducted according to Section 2.7.3. Additionally, the lipid distribution in the liver of experimental rats was also analyzed. The liver tissues were frozen in an optical coherence tomography compound and then stained using Oil Red O dye. The liver tissues were observed under an optical microscopy to check for pathological variations.

#### 2.8.4. 16S rRNA Gene Sequencing and Gut Microbiota Analysis

Total genomic DNA was extracted from the ileal contents. DNA concentration was quantified using a Nanodrop 2000 spectrophotometer (Thermo Fisher Scientific, Waltham, MA, USA), and the DNA quality was verified by 1% agarose gel electrophoresis. The V3-V4 hypervariable region of the 16S rRNA gene was amplified via PCR using primers 338F (5′-ACTCCTACGGGAGGCAGCAG-3′) and 806R (5′-GGACTACHVGGGTWTCTAAT-3′). The PCR products were purified from 2% agarose gels using the AxyPrep DNA Gel Extraction Kit (Axygen Biosciences, Hangzhou, Zhejiang, China), quantified with a Quantus™ fluorometer (Promega Corporation, Madison, WI, USA), and subjected to paired-end sequencing (2 × 250 bp) on the Illumina NovaSeq platform (Illumina, Inc., San Diego, CA, USA). The sequencing data were subjected to a series of processing steps, including splitting, splicing, filtering, and denoising using the Divisive Amplicon Denoising Algorithm (DADA2). Subsequently, sequences sharing ≥97% similarity were grouped into the same operational taxonomic unit (OTU) using UPARSE software (version 11.0). All analyses were conducted based on this data processing pipeline. Then, beta diversity analysis was conducted using Non-metric Multidimensional Scaling (NMDS) to assess compositional differences in ileal microbiota communities across the experimental groups. Additionally, the Linear Discriminant Analysis Effect Size (LEfSe) analysis was performed to identify differentially abundant taxa and to determine group-specific biomarker genera.

#### 2.8.5. Analysis of Short-Chain Fatty Acids (SCFAs) in Ileal Contents

Exactly 50 mg of ileal contents was homogenized in 10 mL of ultrapure water. Following vortex mixing for 60 s, the samples were subjected to ultrasonic extraction for 10 min at 4 °C. The samples were then centrifuged at 12,000× *g* for 10 min at 4 °C. The supernatants were subsequently filtered through 0.22 μm membranes prior to analysis. The SCFA quantification was performed using an ICS-5000+ ion chromatograph (Thermo Fisher Scientific, Waltham, MA, USA) with a Dionex IonPac AS11-HC (250 mm × 4 mm, 4 μm, Thermo Fisher Scientific, Waltham, MA, USA). Mobile phase A was 1.5 mmol/L sodium hydroxide; phase B was 100 mmol/L sodium hydroxide. The metabolites were eluted as follows: 0–25 min, 100% A; 25–25.1 min, 100%–0 A; 25.1–40 min, 0% A; 40–40.1 min, 0–100% A; 40.1–60 min, 100% A. The column temperature was 30 °C, the detector temperature was 25 °C, the injection volume was 3 μL, and the flow rate was 1 mL/min. The concentrations were calculated based on external standard curves, utilizing the peak areas obtained from electrophoretic separation (ES).

### 2.9. Statistical Analysis

Statistical analyses were performed and visualized using GraphPad Prism 9 software (GraphPad Software, Inc.). Quantitative data are presented as mean ± standard deviation (SD). Differences between groups were analyzed using one-way ANOVA followed by Tukey’s HSD post hoc test to correct for multiple comparisons. A value of *p* < 0.05 was considered as statistically significant.

## 3. Results

### 3.1. Nutritional Component Analysis of PSO and Other Vegetable Oils

#### 3.1.1. Fatty Acid Composition and Content

To comprehensively assess the nutritional profile and health benefits of PSO, we analyzed the fatty acid composition of PSO and 15 other vegetable oils (Table 1). The results demonstrated that PSO exhibited superior performance in multiple critical fatty acid parameters, particularly in omega-3 PUFA content. PSO contained 62.79% ALA (C18:3), the highest among all tested oils, significantly surpassing walnut oil (17.99%) and canola oil (12.19%) (*p* < 0.01). This remarkable ALA content positions PSO as a promising vegetable-based source for omega-3 fatty acid supplementation. Moreover, PSO exhibited a low total SFA content of 5.85%, substantially lower than palm oil and rice bran oil, as well as most other common oils. Simultaneously, its total UFA content reached 94.15%, with a balanced ratio of MUFAs to PUFAs.

#### 3.1.2. Total Contents of Phenols and Flavonoids

Phenolic and flavonoid compounds, as critical secondary metabolites in plants, not only serve as the core material basis for the functional activity of vegetable oils but also represent key indicators for evaluating their nutritional quality. This study systematically analyzed the total phenolic and flavonoid contents in 16 representative vegetable oils using the Folin–Ciocalteu method and gallic acid-based standard curve analysis (Table 2). The results demonstrated the high reliability of the total phenolic content determination method, with a linear regression equation of Y = 0.0132X + 0.0137 (R^2^ = 0.998). The total phenolic content ranged from 147 to 511 mg/kg, with sesame oil exhibiting the highest value (511 mg/kg) and grape seed oil exhibiting the lowest value (147 mg/kg) across the 16 oils. For total flavonoid quantification, rutin was used as the reference standard, yielding a linear regression equation of Y = 0.012X + 0.001 (R^2^ = 0.9997). The flavonoid content varied between 45 and 198 mg/kg, with rice bran oil showing the highest level (198 mg/kg) and tea seed oil the lowest (45 mg/kg). Overall, PSO has relatively high levels of total phenols and total flavonoids, indicating that it has a good base of antioxidant compounds.

#### 3.1.3. Composition and Content of Metal Elements

To systematically evaluate the mineral nutritional attributes and elemental safety of PSO relative to 15 other vegetable oils, we quantified 12 metallic elements, Na, Mg, K, Ca, Mn, Fe, Ni, Cu, Zn, As, Cd, and Pb, using ICP-OES. Calibration curves constructed from serial standard solutions exhibited excellent linearity for all target elements (R^2^ ≥ 0.997), confirming the method’s reliability for quantitative analysis.

As shown in Table 3, significant inter-oil variations in elemental composition were observed, likely attributable to differences in botanical origin, agro-environmental conditions, and processing technologies. Regarding macrominerals, Na was highest in sesame oil and lowest in blended oil. Mg peaked in peanut oil, while sunflower oil contained the least. K and Ca also reached maximum levels in peanut oil. Notably, K was undetectable in both sunflower oil and PSO. For trace elements and toxic metals, Mn, Fe, Ni, and Cu were either undetected or present only at trace levels in most oils. Grape seed oil stood out as the only sample containing multiple detectable trace and toxic elements. It contained Zn along with measurable As, Cd, and Pb, and it contained the sole detection of lead among all tested oils. Peanut oil also contained Zn and trace amounts of As and Pb. According to the requirements of GB 2762-2022 [23], the heavy metal content of all 16 types of vegetable oils meets the standard. In contrast, PSO exhibited a distinctive and favorable mineral profile: it contained Na, Mg, Ca, and notably Zn at 10.32 μg/g, which was significantly higher than that of all other oils (*p* < 0.01), highlighting its unique zinc-enriched characteristic. Critically, no detectable levels of As, Cd, or Pb were found in PSO, underscoring its superior elemental safety.

#### 3.1.4. Determination of AV, IV, SV, and PV

Subsequently, the main chemical properties such as AV, PV, IV, and SV of these 16 oils were tested (Table 4). The AV represents the contents of free fatty acids in oil. The result indicated that the AV of PSO was 1.19, and was significantly higher than other oils (*p* < 0.01). A high AV makes it prone to oxidation and reduces the smoke point of the oil. Meanwhile, the IV represents the degree of unsaturation of fats and oils. The result indicated that the IV of PSO was 115.81, which was similar to that of the sunflower oil and soybean oil. And the IV of PSO was significantly high than that of palm oil, avocado oil, tea seed oil and others. The PV is an essential parameter for measuring peroxide and hydroperoxide formation during the initial oxidation phase. The result indicated that the PV of PSO was 0.115, significantly higher than that of linseed oil and sesame oil (*p* < 0.01). The SV indicates the length of the carbon chain of the acid moiety of oil or fat. The greater the percentage of short-chain acids in the oil or fat, the higher the SV. The result indicated that the SV of PSO was 174.91, which was similar to that of the rapeseed oil and olive oil, and was significantly higher than that of tea seed oil and rice bran oil (*p* < 0.01).

### 3.2. Acute Oral Toxicity Testing Results

#### 3.2.1. The Results of General Observations

No mortality was observed in either the control group or any of the PSO-treated groups. All mice exhibited normal behavior in terms of activity, food intake, water consumption, and excretion. Their fur appeared healthy, with normal coloration and a glossy sheen, and they maintained good mental status during the experiment, showing no signs of toxicity.

#### 3.2.2. Histopathological Changes in Liver and Kidney Tissues

**Figure 1:**
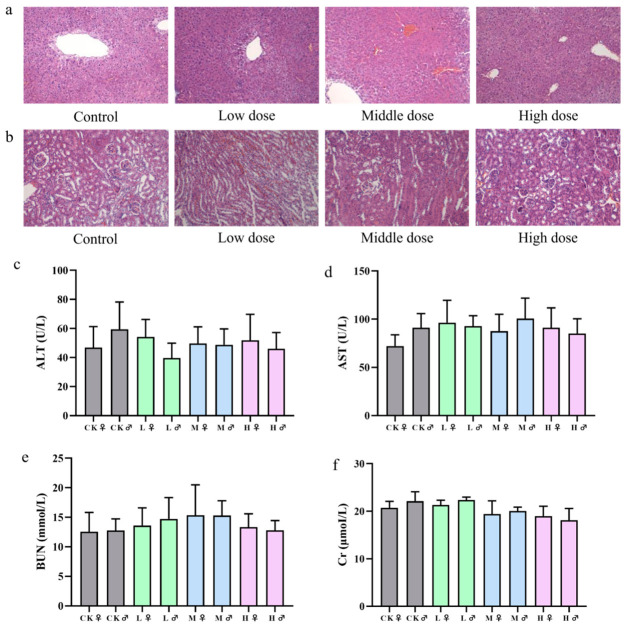
Effects of acute toxicity of PSO on the liver and kidneys (*n* = 10). (**a**) Liver tissue HE staining; (**b**) kidney tissue HE staining; (**c**) serum ALT levels; (**d**) serum AST levels; (**e**) serum BUN levels; (**f**) serum Cr levels. CK: Control group; L: Low dose group; M: Medium dose group; H: High dose group; ♀: Female; ♂: Male.

The acute toxicity test of PSO was performed after seven days of administration; mice were euthanized by cervical dislocation. Liver and kidney tissues were collected and subjected to H&E staining, with representative images shown in Figure 1a,b. H&E staining results revealed that the hepatic architecture was well preserved in both the control and all PSO dose groups (Figure 1a). The liver lobules were clearly demarcated, with hepatocytes arranged radially around the central vein. Similarly, renal tissues exhibited normal morphology in that no signs of edema were observed, renal tubular epithelial cells were orderly aligned, glomeruli maintained a typical structure, and no apparent inflammatory cell infiltration was detected (Figure 1b). These findings indicated that short-term PSO administration at the tested doses did not induce histopathological alterations in the liver or kidneys.

#### 3.2.3. Liver and Kidney Biomarker Testing

As is shown in Figure 1c,d, no statistically significant differences in serum ALT and AST were observed between the control and all PSO-treated groups (*p* > 0.05). Furthermore, ALT and AST levels remained within the normal physiological reference ranges, with no consistent elevation or reduction detected among all the groups. These findings indicated that PSO administration with the tested dosages and the time of duration did not induce measurable hepatotoxicity in mice. Moreover, the result of renal function assessment revealed that the levels of BUN and Cr values fell within the normal physiological range, with minimal variation and no dose-dependent trends between the control and PSO-treated groups (*p* > 0.05) (Figure 1e,f). Collectively, these results suggested that PSO intervention did not compromise renal function in mice.

### 3.3. The Hypolipidemic Effect of PSO and the Changes in Gut Microbiota

#### 3.3.1. PSO Ameliorated Biochemical Indicators Related to HFD

**Figure 2:**
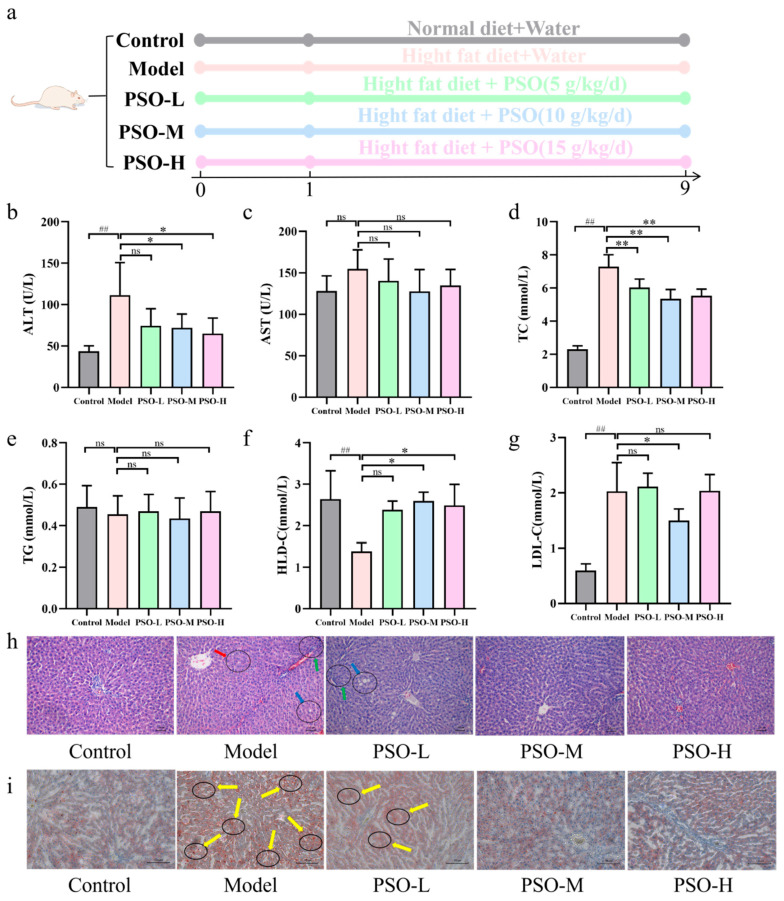
The hypolipidemic effect of PSO and liver pathology in rats fed a high-fat diet (*n* = 10). (**a**) Animal experimental design; (**b**) serum ALT level; (**c**) serum AST levels; (**d**) TC levels; (**e**) TG levels; (**f**) HDL-C levels; (**g**) LDL-C levels; (**h**) representative image of H&E staining of liver tissues (scale: 100 μm), in which blue arrows indicate steatosis, red arrows indicate hepatocyte degeneration, and green arrows indicate inflammatory cell infiltration. (**i**) Representative image of Oil Red O staining of liver tissues (scale: 100 μm), and yellow arrows indicate lipid droplets. ^##^ *p* < 0.01 compared with the model group; ** *p* < 0.01, * *p* < 0.05 compared with the model group; ns: no significant difference.

In this study, HFD-induced hyperlipidemia rat models were established to investigate the pharmacological effects of PSO. Rats were fed a HFD for 8 weeks, followed by daily oral gavage administration of PSO at doses of 5, 10, and 15 g/kg/d (Figure 2a). Compared with the control group, the levels of ALT were significantly increased in the model group (*p* < 0.01), as the hyperlipidemia model may induce hepatic oxidative stress and even steatosis, causing an increase in ALT levels. Meanwhile, compared with the model group, ALT levels were decreased in the PSO-M and PSO-H treatment groups, indicating an improvement in liver function indicators. However, the levels of AST were not significant changed among these groups (Figure 2b,c). In addition, the related indexes of lipid metabolism were also determined. Compared with the control group, the levels of HDL-C were significantly decreased (*p* < 0.01) and the levels of TC and LDL-C were significantly increased in the model rats (*p* < 0.01), indicating successful modeling (Figure 2d–g). In particular, the levels of TC and LDL-C were decreased (*p* < 0.05, *p* < 0.01) and the levels of HDL-C were significantly increased (*p* < 0.05) in the PSO-M group. Thus, these results indicated that the intervention of PSO can affect the levels of TC, LDL-C and HDL-C in hyperlipoidemia to some extent.

#### 3.3.2. PSO Improved the Morphology of the Liver

The morphological changes in liver tissues in each group are shown in Figure 2h. In the control group, the liver cells of rats were neatly arranged in an orderly manner and had a normal size, and the hepatocytes showed no obvious lesions. On the contrary, the model group showed the nucleus was fragmented and the liver tissue structure was disorganized. After treatment with PSO, the morphology of the liver cells was improved, the intercellular space became narrower, few vacuolar lesions of hepatocytes were seen and the structure of the liver cells tended to be normal. To further evaluate the effects of the PSO on alleviating lipid oil droplet distribution, the Oil Red O staining was performed on liver in HFD-fed rats (Figure 2i). The result showed that PSO improved hepatic steatosis and lipid accumulation in liver. Thus, PSO can alleviate pathological damage and reduce lipid accumulation in the liver induced by a HFD.

### 3.4. PSO Improved the Composition of Gut Microbiota

**Figure 3:**
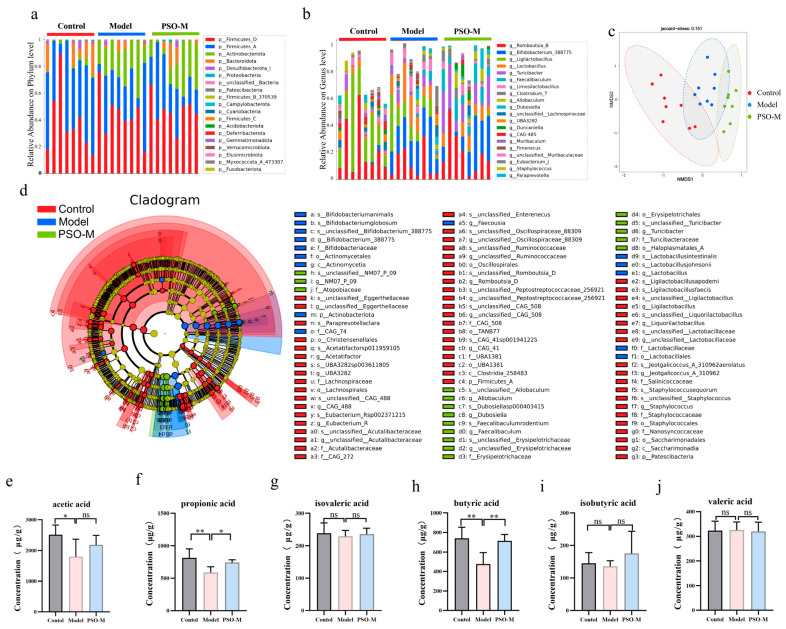
(**a**) Heatmap analysis of phylum and (**b**) genus level of intestinal microbiota in control, model and PSO groups; (**c**) the NMDS plot was created and LEfSe analyses were performed using the default screening thresholds of LDA score > 3.0 and *p* < 0.05. (**d**) Cladogram presented in this study. Effect of PSO on intestinal SCFA concentrations in HFD-induced rats. (**e**) Acetic acid, (**f**) propionic acid, (**g**) isobutyric acid, (**h**) butyric acid, (**i**) isovaleric acid, (**j**) valeric acid. ** *p* < 0.01, * *p* < 0.05 compared with the model group, ns: no significant difference.

A HFD is considered one of the main risk factors implicated in disturbing gut microbiota balance, further promoting the growth of Gram-negative bacteria that produce lipopolysaccharides and aggravating the release of inflammatory factors [24]. We performed 16S rRNA high-throughput sequencing on microbial samples collected from the ileum of the control, model, and PSO-M groups (Figure 3). In general, the microbial composition of the different groups of rats was similar at the phylum and genus level, but distributed in different proportions. The top 20 representative species were selected for ranking based on abundance at the phylum and genus level (Figure 3a,b). β diversity analysis using NMDS demonstrated clear separation of gut microbial communities among the different groups (Figure 3c). Notably, PSO intervention significantly modulated the composition of the gut microbial in rats (Figure 3d,e). Conversely, the relative abundance of various taxa within the *Bacteroidota* phylum, *Proteobacteria* phylum, *Acidobacteriota* phylum, *Romboutsia_B* genus, *Faecalimonas* genus, *Duncaniella* genus, and *Paraprevotella* genus were reduced in the model groups, and the *Actinobacteriota* phylum, *Cyanobacteria* phylum, *Firmicutes_C* phylum, *Deferribacterota* phylum, *Myxococcota*_A_473307 phylum, *Bifidobacterium*_388775 genus, *Limosilactobacillus* genus, and *Anaerotignum*_189125 genus were reduced in the PSO-M group.

To identify differentially abundant gut microbial taxa between these groups, we performed LefSe analysis from the phylum to genus level. Taxa with an LDA score > 3.0 and *p* < 0.05 were identified as key discriminators (Figure 3d). Specifically, Bifidobacterium taxa (e.g., *s__Bifidobacterium animalis* and *s__Bifidobacterium globosum*) and some Lactobacillales (e.g., at the family level, *f__Lactobacillaceae*) exhibited higher relative abundance in the control group, and the abundance of these beneficial microorganisms was significantly reduced in the model group but showed recovery in the PSO-M group. Conversely, the model group displayed a significantly higher abundance of certain unclassified taxa, such as s__unclassified_Oscillospiraceae_88309. These inter-group differences in genus-level abundance were also visualized in the heatmap (Figure 3d).

Given that gut dysbiosis is often associated with decreased production of SCFAs, we quantified the levels of six SCFAs (acetic, propionic, isobutyric, butyric, isovaleric, and valeric acids) in the rats’ ileum (Figure 3e–j). All six SCFAs were found at higher concentrations in the control group compared to the model group. Specifically, the levels of acetic, propionic, and butyric acids were significantly lower in the model group than the control group. Importantly, the levels of propionic and butyric acids were significantly increased in the PSO-M group relative to the model group, suggesting a restorative effect of PSO on SCFA production.

## 4. Discussion

Vegetable oils are increasingly recognized as an indispensable ingredient for their health benefits and nutritional properties, as they contain fatty acids and micronutrients such as flavonoids, phytosterols, tocopherols, carotenoids, and phenolics, and beneficial metal elements [24]. The functional value of vegetable oils not only includes their sensory components as a cooking medium and supply of energy, but also the ability to scavenge free radicals, reduce blood cholesterol, and help prevent chronic diseases [25,26]. For example, intake of sesame oil and PSO can help reduce blood glucose levels, lower oxidative stress, and improve biomarkers of liver and kidney function in patients with type 2 diabetes mellitus, possibly due to the bioactive components in these oils, including fatty acids, tocopherols, and phytosterols [27]. In this study, we provided a comprehensive, multidimensional assessment of PSO’s nutritional superiority, elemental safety, oxidative characteristics, and biological functionality, particularly its role in modulating lipid metabolism and gut microbiota under a HFD.

The fatty acid composition defines the nutritional value of vegetable oils. Based on their chemical structure, dietary fatty acids are primarily categorized into SFAs, MUFAs, and PUFAs [28]. SFAs have complex biological effects, and excessive intake is associated with elevated levels of blood cholesterol, triglycerides, and LDL, thereby increasing the risk of cardiovascular diseases [29]. MUFAs are generally considered to have a neutral or modestly beneficial effect on blood cholesterol profiles [30]. In contrast, PUFAs are known to exert beneficial effects against obesity, fat accumulation, and inflammation [31]. In addition, PUFAs can be further divided into two important series: ω-3 and ω-6. ALA belongs to ω-3 polyunsaturated fatty acids [29]. This study determined PSO contains ALA at a concentration of 62.79%, consistent with previous reports [32] and significantly higher than that of commonly used oils such as walnut oil. Previous studies have indicated that PSO UFAs represent 91.94% and SFAs comprise 8.06% of its total content [33]. In this study, the contents of UFAs and SFAs were 83.88% and 16.12%, respectively. This variation may be caused by different extraction methods. For example, microwave treatment of perilla seeds has been shown to improve the quality of perilla oil, including increases in fatty acid content and phytosterols, as well as enhancements in oxidative stability and DPPH radical scavenging activity [34]. Furthermore, nutritional quality indices based on fatty acid composition revealed that PSO possesses the highest PUFA/SFA ratio, underscoring its superior potential in promoting cardiovascular health and reducing the risk of atherosclerosis compared to other plant oils.

In addition, to determine the contents of fatty acids in PSO and 15 representative vegetable oils, the contents of total phenolics and flavonoids were also detected in this study. Phenolic and flavonoid compounds are important for the oxidative stability of PUFAs in vegetable oils, attracting considerable research interest due to their diverse bioactivities [35]. Chen et al. developed a bio-based multifunctional molecule through the conjugation of 4-aminodiphenylamine with various phenolic acids; this novel compound outperformed commercial phenolic antioxidants in several vegetables [36]. Moreover, flavonoids and their analogs have been shown to modulate immune cell functions through multiple pathways, influencing inflammatory responses and immune regulation [37]. In addition, the total phenolic content in oils varies greatly depending on the oil source and extraction methods, such as heat treatment, which may disrupt plant cell structures and promote the release of these compounds [38]. Our findings revealed that PSO contains substantial levels of total phenolics and total flavonoids, highlighting its value as a rich natural source of phenolic phytochemicals and underscoring its promising potential for development as a functional oil in nutraceutical and food applications.

Metal elements generally exist in vegetable oil, and play important roles in human life. Na, Mg, K, and Ca are the main macroelements, accounting for about 99% of the total metal ions in the human body, and they participate in physiological processes such as cell synthesis, osmotic balance, and metabolic maintenance [39]. Our analysis revealed that PSO contained low levels of Na, Mg, and Ca, with K below the detection limit, indicating that it cannot serve as a significant source of these macroelements and should be complemented by other foods in the diet to ensure nutritional adequacy. In contrast, PSO was notably rich in Zn, with a concentration of 10.32 μg/g. As an essential cofactor for DNA synthesis and the metabolism of proteins, lipids, carbohydrates, and nucleic acids, Zn plays a vital role in normal growth, development, and immune function. Critically, no detectable levels of the toxic heavy metals As, Cd, or Pb were found in PSO, confirming the absence of environmental contamination during processing and underscoring its high safety profile with respect to heavy metal exposure. Heavy metal contamination from the planting soil and the processing mode of PSO comes from the planting environment, raw material harvesting, warehousing and transportation, as well as oil processing. Therefore, this effectively guarantees the quality and safety of PSO.

Moreover, the main chemical properties of acid value, iodine value and saponification value were measured. The acid value reflects the concentration of free fatty acids and serves as a critical indicator of hydrolytic degradation and oxidative susceptibility, and higher values imply greater vulnerability to rancidity [40]. The elevated acid value observed in PSO was likely attributable to its production via unrefined cold-pressing without deacidification, a process that preserved native bioactive compounds but retained higher levels of free fatty acids. Lipid oxidative stability is intrinsically linked to fatty acid unsaturation. The iodine value, a well-established measure of unsaturation, increases with the number of double bonds and higher values indicate greater unsaturation and consequently reduced oxidative stability [41,42]. The markedly elevated iodine and peroxide values in PSO were consistent with its exceptionally high ALA content, which rendered the oil more susceptible to oxidation during storage. This underscores the need for appropriate packaging and storage conditions, such as light protection and refrigeration, to maintain the quality of PSO [43]. The saponification value inversely correlates with the average molecular weight of fatty acids: higher values correspond to shorter fatty acid chains [42]. The narrow range of saponification values across the 16 oils suggested similarity in overall fatty acid chain length.

In order to further evaluate the safety of PSO, acute toxicity testing was performed. Acute toxicity testing serves as a cornerstone in the safety evaluation of food-derived compounds, pharmaceuticals, and functional ingredients, directly informing dose selection for clinical or dietary applications [44,45]. In this study, a single oral gavage administration of PSO at the maximum tested dose (10 g/kg) revealed no mortality, behavioral abnormalities, or histopathological alterations in liver and kidney in mice. Serum biomarkers of hepatorenal function remained within physiological ranges across all treatment groups. According to the OECD Guideline for Testing of Chemicals (TG 420) classification criteria, 10 g/kg of PSO was categorizing as “non-toxic” under acute exposure conditions [46]. Previous results also showed no significant treatment-associated toxicity or mortality at PSO dosages up to 50 g/kg in KM mice, 20 g/kg in Wistar rats and 3 g/kg in Beagle dogs during the acute and sub-chronic 90-day oral toxicity experiment [47]. Thus, these results also provided critical toxicokinetic reference values for designing dietary interventions targeting PSO intake without compromising safety, highlighting PSO’s exceptional omega-3 PUFA content and its antioxidant-rich phytochemical profile.

At the same time, we evaluated the lipid-modulating effects of PSO and its impact on gut microbiota in animal experiments. In this study, the use of PSO increased the abundance of *Bacteroidota*, thereby reducing the *Firmicutes*-to-*Bacteroidota* ratio in the ileum of hyperlipidemic rats. This result is similar to the effects observed with sunflower seed oil [48] and tea seed oil [49]. Additionally, PSO intervention decreased the abundance of *Firmicutes* while increasing the abundance of *Actinobacteria*, both of which are involved in lipid homeostasis regulation [50]. Notably, PSO intervention significantly increased the abundance of *Faecalimonas* and *Paraprevotella* in HFD-induced rats. In a healthy gut, *Faecalimonas* is known to produce butyric acid, which helps regulate intestinal pH and maintain the internal environment [51]. *Paraprevotella*, belonging to the phylum *Bacteroidota* and classified within the order *Bacteroidales* and family *Prevotellaceae*, is known to produce propionic acid and butyric acid [51]. Additionally, SCFAs not only inhibit hepatic lipid deposition, but they may also activate the AMPK pathway in adipose tissue, promoting lipolysis and increasing the influx of circulating free fatty acids to the liver [52,53]. Furthermore, butyric acid inhibits the inflammatory response and restores hepatic insulin sensitivity by suppressing the expression of PPAR [54,55]. Consistently, our findings show that PSO intervention increases propionic and butyric acid levels in the ileum of HFD-induced rats, which may be associated with an enhanced abundance of *Faecalimonas* and *Paraprevotella*, major producers of SCFAs.

Although this article has explored the nutritional quality, elemental safety, and the changes in gut microbiota in hyperlipidemia of PSO, the specific active ingredients responsible for its lipid-modulating effects remain unclear. In the future study, we will thoroughly investigate the difference in hypolipidemic activity between ALA, phenolic and flavonoid compounds, and other representative vegetable oils, to fully clarify the potential mechanism and its regulatory role in gut microbiota. This study will provide a solid foundation for PSO as a daily supplement for preventing lipid metabolism disorders.

## 5. Conclusions

This study provided the first systematic, multidimensional evaluation of PSO within a “composition–safety–function–mechanism” framework. These findings demonstrated that PSO possessed a high ALA content and abundant phenolic and flavonoid compounds, which collectively underpin its remarkable cardiovascular and metabolic health benefits. Safety assessments revealed no detectable levels of heavy metals such as As, Cd, or Pb, while Zn was present in notable amounts. Acute toxicity testing showed no abnormalities in serum hepatic and renal biomarkers or histopathology in mice even at a dose of 10 g/kg. Animal experiments further confirmed that PSO effectively ameliorated HFD-induced dyslipidemia and significantly reducing serum TC and LDL-C while increasing HDL-C. Moreover, PSO increased the abundance of beneficial bacteria including *Bacteroidota*, restored gut microbiota balance, and enhanced the production of short-chain fatty acids, particularly propionic and butyric acids. Thus, PSO may be a potential nutritional approach for dietary management and the prevention of hyperlipidemia.

## Figures and Tables

**Table 1:** Fatty acid contents of 16 vegetable oils.

Samples	Fatty Acid Contents (%)										SFA (%)	UFA (%)
	C12:0	C14:0	C16:0	C16:1	C18:0	C18:1	C18:2	C18:3	C20:0	C20:1		
PO	0.21	1.13	55.97	0.23	6.23	30.07	6.61	0.20	0.51	0.18	63.21	36.79
SO	-	-	15.51	0.19	9.65	34.75	38.00	0.53	1.03	0.34	26.20	73.80
WO	-	-	11.70	0.11	4.57	15.19	50.16	17.99	-	0.29	16.27	83.73
AO	-	-	11.71	2.30	5.26	64.66	15.05	0.26	0.41	0.36	17.38	82.62
FO	-	-	7.71	0.10	6.08	13.37	9.56	62.77	0.21	0.20	14.00	86.00
TSO	-	-	10.77	0.21	4.90	74.18	8.56	0.38	0.35	0.64	16.02	83.98
GSO	-	-	13.53	0.16	8.48	13.54	61.82	1.77	0.40	0.32	22.41	77.59
SunO	-	-	11.77	0.20	6.66	24.97	55.46	0.22	0.45	0.28	18.88	81.12
OO	-	-	20.26	1.51	5.92	64.38	5.82	0.99	0.71	0.41	26.89	73.11
SBO	-	-	19.09	0.18	7.65	19.91	43.89	8.16	0.71	0.41	27.45	72.55
CO	-	-	21.39	0.17	3.18	23.02	49.60	1.47	0.64	0.52	25.22	74.78
BO	-	-	13.56	0.24	5.61	31.71	37.88	9.42	0.76	0.82	19.93	80.07
RBO	-	0.47	29.08	0.25	2.83	34.18	29.82	1.90	1.02	0.94	33.24	66.76
PeaO	-	-	19.51	0.11	6.79	38.90	30.13	0.00	2.73	1.82	29.03	70.97
RO	-	-	8.12	0.37	3.33	55.60	16.06	12.19	1.04	3.29	12.49	87.51
PSO	-	-	3.21	0.12	2.47	15.38	15.52	62.79	0.17	0.34	5.85	94.15

This table presents the contents of major fatty acids in 16 vegetable oils, including palm oil (PO), sesame oil (SO), walnut oil (WO), avocado oil (AO), flaxseed oil (FO), tea seed oil (TSO), grape seed oil (GSO), sunflower oil (SunO), olive oil (OO), soybean oil (SBO), corn oil (CO), blended oil (BO), rice bran oil (RBO), peanut oil (PeaO), rapeseed oil (RO), and perilla seed oil (PSO). The main fatty acids were lauric acid (C12:0), myristic acid (C14:0), palmitic acid (C16:0), palmitoleic acid (C16:1), stearic acid (C18:0), oleic acid (C18:1), linoleic acid (C18:2), α-linolenic acid (C18:3), arachidic acid (C20:0), and eicosenoic acid (C20:1). “-” in the table indicates an acid was not detected or below the detection limit. SFAs (saturated fatty acids) = C12:0 + C14:0 + C16:0 + C18:0 + C20:0; UFAs (unsaturated fatty acids) = C16:1 + C18:1 + C18:2 + C18:3 + C20:1.

**Table 2:** The contents of the total phenols and flavonoids in 16 vegetable oils (*n* = 3).

Sample	Total Phenols (mg/kg)	Total Flavonoids (mg/kg)
PO	172.56 ± 3.78 **	58.89 ± 10.08 **
SO	511.50 ± 43.17 **	91.67 ± 3.01 **
WO	154.68 ± 14.90 **	122.22 ± 43.50 **
AO	157.56 ± 4.20 **	59.72 ± 14.05 **
FO	155.14 ± 6.73 **	93.61 ± 16.92 **
TSO	149.38 ± 5.42 **	45.56 ± 3.94 **
GSO	147.26 ± 19.73 **	55.56 ± 6.94 **
SunO	167.26 ± 13.83 **	54.72 ± 7.47 **
OO	171.20 ± 17.73 **	62.50 ± 6.30 **
SBO	162.86 ± 23.66 **	65.56 ± 12.92 **
CO	179.38 ± 25.04 **	87.78 ± 14.30 **
BO	167.71 ± 27.76 **	85.83 ± 10.10 **
RBO	312.86 ± 31.89 **	198.06 ± 19.83 **
PeaO	228.92 ± 19.85 **	48.33 ± 6.29 **
RO	281.50 ± 40.84 **	62.22 ± 9.14 **
PSO	254.23 ± 6.36	69.16 ± 23.76

** *p* < 0.01 versus the PSO.

**Table 3:** The metal element contents in 16 vegetable oils (μg/g, *n* = 3).

Sample	Na	Mg	K	Ca	Mn	Fe	Ni	Cu	Zn	As	Cd	Pb
PO	53.89 ± 1.24 **	10.48 ± 0.30 **	4.76 ± 0.86 **	13.71 ± 0.88 *	-	-	-	0.02 ± 0.01	1.78 ± 0.05 **	-	-	-
SO	82.00 ± 5.18	17.69 ± 0.32	3.41 ± 1.07 **	7.35 ± 0.66 **	-	-	-	-	-	-	-	-
WO	79.86 ± 3.67 *	17.41 ± 0.56	2.10 ± 0.70 **	15.41 ± 0.89 **	-	-	-	-	-	0.04 ± 0.01 **	-	-
AO	49.90 ± 2.26 *	10.30 ± 0.07 **	3.45 ± 0.75 **	19.78 ± 0.66 **	-	-	-	-	-	0.05 ± 0.03 **	-	-
FO	63.55 ± 1.45	14.01 ± 0.12 *	2.69 ± 0.98 **	16.01 ± 0.33 **	-	-	-	-	-	-	-	-
TSO	57.90 ± 2.79	17.95 ± 0.16	4.71 ± 0.64 **	17.73 ± 0.31 **	-	-	-	-	-	0.02 ± 0.01 **	-	-
GSO	51.39 ± 4.20	28.47 ± 0.32 **	3.33 ± 0.40 **	67.97 ± 1.47 **	-	-	-	-	0.77 ± 0.07 **	0.08 ± 0.02 **	0.03 ± 0.01 **	0.65 ± 0.01 **
SunO	46.59 ± 5.41	8.17 ± 0.15 **	-	6.43 ± 0.45 **	-	-	-	-	-	0.01 ± 0.00 **	-	-
OO	61.21 ± 2.14	13.27 ± 0.15 *	1.32 ± 0.43 **	10.67 ± 0.42	-	-	-	-	-	0.05 ± 0.00 **	-	-
SBO	56.72 ± 6.00	9.91 ± 0.70 **	2.56 ± 1.24 **	12.21 ± 1.66	-	-	-	-	-	0.17 ± 0.06 **	-	-
CO	45.76 ± 2.34	10.63 ± 0.19 **	2.37 ± 0.85 **	13.57 ± 1.12 *	-	-	-	-	-	-	-	-
BO	42.77 ± 1.77	12.88 ± 0.32 **	0.63 ± 0.15 **	13.03 ± 0.62 *	-	-	-	-	-	0.04 ± 0.01 **	-	-
RBO	51.06 ± 1.97 *	12.16 ± 0.15 **	2.66 ± 0.76 **	11.02 ± 1.18	-	-	-	-	-	-	-	-
PeaO	59.11 ± 2.72 *	41.23 ± 0.30 **	5.34 ± 0.46 **	88.96 ± 0.50 **	-	-	-	-	0.78 ± 0.06 **	0.05 ± 0.02 **	-	0.41 ± 0.01 **
RO	65.18 ± 0.77 **	15.00 ± 0.28 *	3.06 ± 1.42 **	7.99 ± 0.27 *	-	-	-	-	-	-	-	-
PSO	48.33 ± 2.31	17.34 ± 0.38	-	11.19 ± 0.34	-	-	-	-	10.32 ± 0.09	-	-	-
GB 2762-2022							<1000			<100	<200	<80

* *p* < 0.05 and ** *p* < 0.01 versus the PSO; “-” means not detected.

**Table 4:** The measurement of AV, IV, PV, and SV in 16 vegetable oils (*n* = 3).

Sample	Acid Value (mg/g)	Iodine Value (g/100 g)	Peroxide Value (g/100 g)	Saponification Value (mg/100 g)
PO	0.19 ± 0.02 **	43.33 ± 1.92 **	0.006 ± 0.001 **	178.64 ± 2.62
SO	0.73 ± 0.04 **	107.96 ± 1.21	0.007 ± 0.001 **	165.03 ± 2.42
WO	0.25 ± 0.05 **	104.50 ± 1.58 *	0.054 ± 0.002 **	159.15 ± 12.51
AO	0.19 ± 0.02 **	43.43 ± 4.26 **	0.064 ± 0.006 *	160.50 ± 4.89
FO	0.14 ± 0.03 **	112.48 ± 11.74	0.040 ± 0.001 **	169.32 ± 8.42
TSO	0.18 ± 0.03 **	85.69 ± 1.01 **	0.078 ± 0.006	149.53 ± 17.74 **
GSO	0.15 ± 0.01 **	111.07 ± 1.12	0.072 ± 0.001	172.18 ± 0.94
SunO	0.30 ± 0.05 **	114.54 ± 4.03	0.077 ± 0.001	179.17 ± 16.94
OO	0.57 ± 0.02 **	90.67 ± 3.60 **	0.069 ± 0.002 *	172.57 ± 16.05
SBO	0.09 ± 0.00 **	115.98 ± 9.16	0.019 ± 0.001 **	154.20 ± 18.10 *
CO	0.22 ± 0.05 **	119.39 ± 7.83	0.009 ± 0.004 **	161.27 ± 5.12
BO	0.36 ± 0.00 **	85.32 ± 5.78 **	0.033 ± 0.003 **	167.98 ± 12.98
RBO	0.90 ± 0.07 **	89.71 ± 5.13 **	0.052 ± 0.010 **	145.31 ± 17.07 **
PeaO	0.88 ± 0.06 **	93.08 ± 4.11 **	0.046 ± 0.006 **	164.25 ± 4.42
RO	0.51 ± 0.09 **	106.05 ± 6.35 *	0.067 ± 0.010 *	174.56 ± 9.46
PSO	1.19 ± 0.09	115.81 ± 11.26	0.115 ± 0.090	174.91 ± 9.38
GB 2716-2018	<3	-	<0.25	-

* *p* < 0.05 and ** *p* < 0.01 versus the PSO.

## Data Availability

Data will be made available on reasonable request.

## Abbreviations

The following abbreviations are used in this manuscript:

PSO	Perilla seed oil
ALA	α-Linolenic acid
HFD	High-fat diet
TC	Total cholesterol
TG	Triglyceride
LDL-C	Low-density lipoprotein cholesterol
HDL-C	High-density lipoprotein cholesterol
SCFAs	Short-chain fatty acids
PUFA	Polyunsaturated fatty acid
MUFA	Monounsaturated fatty acid
SFA	Saturated fatty acid
ALT	Alanine transaminase
AST	Aspartate transaminase
BUN	Blood urea nitrogen
Cr	Creatinine
ICP-OES	Inductively coupled plasma optical emission spectrometry
AV	Acid value
IV	Iodine value
PV	Peroxide value
SV	Saponification value
SPF	Specific pathogen-free
H&E	Hematoxylin and eosin
